# Mosquito saliva alone has profound effects on the human immune system

**DOI:** 10.1371/journal.pntd.0006439

**Published:** 2018-05-17

**Authors:** Megan B. Vogt, Anismrita Lahon, Ravi P. Arya, Alexander R. Kneubehl, Jennifer L. Spencer Clinton, Silke Paust, Rebecca Rico-Hesse

**Affiliations:** 1 Integrative Molecular and Biomedical Sciences Graduate Program, Baylor College of Medicine, Houston, Texas, United States of America; 2 Department of Molecular Virology and Microbiology, Baylor College of Medicine, Houston, Texas, United States of America; 3 Department of Pediatrics, Baylor College of Medicine, Houston, Texas, United States of America; University of Florida, UNITED STATES

## Abstract

Mosquito saliva is a very complex concoction of >100 proteins, many of which have unknown functions. The effects of mosquito saliva proteins injected into our skin during blood feeding have been studied mainly in mouse models of injection or biting, with many of these systems producing results that may not be relevant to human disease. Here, we describe the numerous effects that mosquito bites have on human immune cells in mice engrafted with human hematopoietic stem cells. We used flow cytometry and multiplex cytokine bead array assays, with detailed statistical analyses, to detect small but significant variations in immune cell functions after 4 mosquitoes fed on humanized mice footpads. After preliminary analyses, at different early times after biting, we focused on assessing innate immune and subsequent cellular responses at 6 hours, 24 hours and 7 days after mosquito bites. We detected both Th1 and Th2 human immune responses, and delayed effects on cytokine levels in the blood, and immune cell compositions in the skin and bone marrow, up to 7 days post-bites. These are the first measurements of this kind, with human immune responses in whole animals, bitten by living mosquitoes, versus previous studies using incomplete mouse models and salivary gland extracts or needle injected saliva. The results have major implications for the study of hematophagous insect saliva, its effects on the human immune system, with or without pathogen transmission, and the possibility of determining which of these proteins to target for vaccination, in attempts to block transmission of numerous tropical diseases.

## Author summary

Mosquito saliva proteins have numerous effects on the immune system, and we describe here the use of mice with a humanized immune system to study the effects of mosquito bites on human cells. Our results show that the number of immune cell types affected is much larger than previously described, and some immune responses to mosquito bites can be detected up until 7 days post-bite. The biological significance of these changes remains to be determined, but it might explain how some pathogens, such as viruses, can spread through the body in these cells, replicate to higher extents, and even remain in some tissues for far longer than detected in blood.

## Introduction

Approximately 750,000 people die of mosquito transmitted diseases each year, including malaria, dengue, West Nile, Zika, and chikungunya fevers [1, 2]. The incidence of these diseases is expected to rise significantly in the next few decades as the host ranges of multiple species of mosquitoes increase due to climate change [3, 4]. Not only do mosquitoes transmit diseases, but they also may increase the severity of the diseases they transmit. In experimental infections of mice, delivery of arboviruses via mosquito bite or via needle injection in conjunction with uninfected mosquito bite results in more severe disease than delivery of virus alone via needle injection [5–9]. In mouse models of infections by malaria and leishmania parasites, mosquito and sandfly saliva have also been shown to enhance infectivity and disease progression [10, 11].

Mosquito saliva is a complex mixture of proteins that allows the mosquito to acquire a blood meal from its host (necessary for egg maturation), by circumventing vasoconstriction, platelet aggregation, coagulation, and inflammation or hemostasis (reviewed in [12]). It is well known that mosquito saliva contains proteins that are immunogenic to humans, and some allergic responses can be very severe [13, 14]. Because mosquito saliva can be immunogenic, it is speculated that mosquito saliva may enhance pathogenicity of arboviruses by manipulating the host’s immune response.

The human immune system can be divided into innate (natural killer (NK) cells, neutrophils, monocytes, macrophages, mast cells, and dendritic cells) and adaptive (T and B cells) arms. Upon activation, innate immune cells release cytokines and chemokines, kill cells via cytotoxic molecules (NK cells only), and phagocytose pathogens, which aids in pathogen destruction and allows specialized cells to process antigens for presentation to T and B cells. Successful T and B cell activation by their cognate antigen induces their differentiation into clonal effector or long-lived antigen specific memory cells both of which are crucial to protect us from recurrent, severe disease (reviewed in [15]).

T cells are often classified based on expression of the CD4 or CD8 co-receptors. Upon activation, CD4 T cells can differentiate into a variety of T helper cells (Th1, Th2, etc.), each with unique functions, whereas CD8 T cells become cytotoxic T lymphocytes (CTLs) [16, 17]. A small proportion of T cells outside of the thymus express both CD4 and CD8. These double positive (DP) T cells arise from CD4 or CD8 T cells that upregulate expression of the other co-receptor in the periphery through an unknown mechanism [18]. DP T cells can have similar, albeit more exaggerated, functions to CTLs [18–23]; moreover, they can regulate or exacerbate inflammation through the production of cytokines, including IL-10, IL-4, and IFNγ [18, 24, 25]. Another type of T cell that performs similar functions to DP T cells is the natural killer T (NKT) cell. These cells, which develop in the thymus and express a rearranged T cell receptor and NK cell markers, can be cytotoxic. Activated NKT cells are also potent and rapid cytokine producers and can polarize immune responses through the production of large amounts of IFNγ, IL-4, and/or IL-17A [26–28].

Immune responses can be classified according to the type of helper T cell involved; the two canonical immune responses are the Th1 response and the Th2 response. The Th1 response typically involves a cell-mediated response to eradicate intracellular pathogens. Antigen presenting cells activated by intracellular pathogens produce the cytokines IL-12 and IL-18. These cytokines stimulate naïve CD4 T cells to differentiate into Th1 T cells, which produce the cytokines IFNγ, IL-2, and TNFβ and stimulate the activation of CTLs. Th1 cells also stimulate B cells to produce specific IgG isotypes that mediate effector functions against intracellular pathogens [29]. The Th2 response typically involves a strong humoral response to eradicate extracellular pathogens and parasites. When overactive, a Th2 response can cause allergies or asthma. During a Th2 response, activated mast cells or T cells produce the cytokine IL-4, which stimulates naïve CD4 T cells to become Th2 cells. These Th2 cells produce more IL-4 as well as the cytokines IL-5, IL-6, IL-9, IL-10, and IL-13, which together, inhibit phagocytic inflammation and promote the proliferation and survival of mast cells and other cells needed for the clearance of extracellular pathogens. Th2 cells also stimulate B cells to produce antibodies of the IgE isotype. Th1 and Th2 responses are not necessarily mutually exclusive, as mixed Th1/Th2 immune response can occur; however, cytokines produced in each response do actively inhibit production of cytokines and processes involved in the other response (reviewed in [17, 30, 31]).

In this study, we sought to investigate the effects of mosquito saliva on the human immune system. Previous studies have addressed this phenomenon but used mouse models genetically predisposed to a Th1 (C57BL6 mice) or Th2 (BALB/c mice) response, and/or used mosquito salivary gland extracts instead of secreted mosquito saliva or mosquito bites [32, 33]. These studies have produced conflicting results as to the anti-inflammatory and viral enhancing properties of saliva proteins [13]. Furthermore, these studies did not use detailed analyses of cellular responses to mosquito bites or use mouse models that mimic human infection [34, 35]. It is clear that mouse models of human disease do not replicate many innate and adaptive immune responses [36], so we sought to create a model in which we could measure the human cytokine and cellular responses, *in vivo*. Thus, we have developed a humanized mouse model of mosquito biting, to study the specific effects of mosquito saliva on the pathogenesis of mosquito-borne viruses. Our results are the only to measure the effects of mosquito bite on specific components of the human immune system, especially early after saliva injection, and they indicate long-lasting effects of these components, up to 7 days in human skin and bone marrow cells.

## Materials and methods

### Mosquito rearing

*Aedes aegypti* (Rockefeller) mosquitoes were obtained from BEI resources as eggs (MRA-734). Mosquitoes were maintained under standard insectary conditions (~28°C, 80% relative humidity) with a 12-hour light/dark cycle maintained by the Philips Hue Smart Lighting system. Larvae were raised in water pans and fed on a mixture of ground rabbit chow (Purina)-liver powder (Bio-Serv)-yeast (Bio Serv) in a 4:1:1 ratio, *ad libitum*. Emerged mosquitoes were moved to mesh cages and fed on 10% sucrose (Sigma) solution *ad libitum*. Colony maintenance was performed by feeding mosquitoes on anesthetized C57/BL6 mice (IACUC AN-6151). In the subsequent days following blood feeding, eggs were collected, desiccated, and stored for a maximum of 6 months.

### Mosquito salivation

Mosquito saliva was collected from female *Aedes aegypti* Rockefeller mosquitoes 4–5 days post-eclosion. Adult female mosquitoes were immobilized by removal of wings and legs. The proboscis was inserted into a 10μL microcapillary tube (Drummond) containing microscopy-grade immersion oil (Zeiss). The mosquitoes were allowed to salivate into the oil at insectary conditions for 45 minutes. After salivation, the mosquitoes were confirmed to have salivated and be alive as evidenced by saliva droplets in the oil, and response to physical stimulus, respectively. Saliva was extracted immediately from the oil by pooling the salivations and adding one volume of cold phosphate buffered saline (PBS). The salivation/PBS mixture was pulse vortexed and centrifuged at 10,000g for 5min. The aqueous layer was removed and the protein concentration was quantified using the BCA assay (Pierce).

### Isolation and stimulation of human PBMCs

Source leukocytes were obtained from the Gulf Coast Regional Blood Center (Houston, TX). PBMCs were isolated via density centrifugation using Leucosep centrifuge tubes (Grenier Bio-one) and lymphocyte separation media (Corning) according to manufacturers’ protocols. To remove any residual red blood cell contamination, PBMC pellets were then treated with red blood cell lysis solution (eBiosciences) according to manufacturer’s instructions. PBMCs were resuspended in RPMI 1640 media with 10% fetal bovine serum (FBS) and were plated at 1x10^6^ cells/well in a 24 well plate. PBMCs were stimulated with mosquito saliva (2μg salivary proteins per well), or the equivalent of 4 mosquito bites [37], to correspond with the number of infected mosquito bites required to produce dengue fever in humanized mice [7], and lipopolysaccharide (1mg/L media; Sigma), pokeweed mitogen (5mg/L media; Sigma), or untreated media. Supernatant samples were collected daily for five days post stimulation to assess cytokine production. On day five post stimulation, all PBMCs were assessed via flow cytometry.

### Production of humanized mice

Humanized mice were engrafted as previously described [7]. Briefly, male and female NSG breeders were obtained from The Jackson Laboratory, and mice were bred in the Transgenic Mouse Facility at Baylor College of Medicine. One day post birth, each pup from these breedings was sublethally irradiated with 100 centigrays and intrahepatically injected with 3x10^5^ CD34+ stem cells. These stem cells were isolated from human umbilical vein cord blood from either the University of Texas MD Anderson Cord Blood Bank (Houston, TX) or the Texas Cord Blood Bank (San Antonio, TX) using the Dynabeads CD34 positive selection kit (Invitrogen) following the manufacturer’s instructions. Levels of engraftment of human hematopoietic cells were tested 6 to 8 weeks later using flow cytometry to target human and mouse CD45+ cells (S1 Table).

### Mosquito biting of Hu-NSG mice

Mosquito biting of reconstituted humanized mice was carried out as previously reported [7], although with uninfected mosquitoes. In short, 4 to 7 days post-emergence, female mosquitoes were starved for 24 hours in dram vials (4–6 mosquitoes per vial) capped in a fine, white polyester mesh (Bio-Serv). Dram vials were kept at insectary conditions (28°C, 80% humidity) for the duration of the 24-hour starving. Mosquitoes were then transferred to a BSL3 facility, and the dram vials were held against a footpad of anesthetized, humanized mice, allowing the mosquitoes to feed (IACUC AN-6151). A “bite” was defined visually by mosquito engorgement and did not include probing; approximately 4 bites total occurred for each mouse, on both footpads. This number was chosen based on our previous studies demonstrating that 4 infected mosquitoes are required to bite each humanized mouse to consistently produce dengue fever [7].

### Tissue collection and processing

Six hours, 24 hours, or 7 days post mosquito bite, mice were humanely euthanized via isoflurane overdose. Upon cessation of breathing, mice were exsanguinated via intracardiac bleed. Blood was stored in heparin treated and untreated microcentrifuge tubes for further processing. Skin from rear footpads was removed using surgical scissors and stored separately in PBS with 2% FBS (PBS/FBS) and 5μg/mL collagenase. Spleens and femurs were also removed from each mouse and stored separately in PBS/FBS.

Blood stored in untreated tubes was allowed to clot at room temperature for at least thirty minutes. The clotted blood was then centrifuged at 1500g for ten minutes at 4°C. Following this centrifugation, serum was transferred to new microfuge tubes and stored at -70°C until used in multiplex cytokine bead array assay. Blood stored in heparinized tubes were transferred to 50mL conical tubes. Red blood cells were lysed using RBC lysis solution (eBioscience) according to the manufacturer’s protocol. The remaining white blood cell pellet was resuspended at 1x10^4^ to 1x10^6^ cells/mL in PBS/FBS. These cells were stored at 4°C until stained for flow cytometry analysis.

Skin footpads were cut into small pieces and incubated in PBS/FBS and 5mg/mL collagenase at 37°C for 1 hour. Following digestion, skin pieces were ground over a 40μm cell strainer into a 50mL conical tube. Skin cells were washed twice in PBS/FBS and resuspended at 1x10^4^ to 1x10^6^ cells/mL in PBS/FBS.

Spleens were burst by grinding between two frosted microscope slides. Spleen contents were then ground over a 40μm strainer into a 50mL conical tube. Red blood cells were lysed using RBC lysis solution (eBioscience) according to the manufacturer’s protocol. Remaining cells were resuspended at 1x10^6^ – 1x10^7^ cells/mL in PBS/FBS.

Bone marrow was flushed out of femurs using a 25G needle filled with PBS/FBS. Marrow was ground over a 40μm strainer into a 50mL conical tube. Red blood cells were lysed and remaining cells were resuspended as with the spleen cells.

### Flow cytometry

Blood, bone marrow, skin, and spleen cells from hu-NSG mice and stimulated human PBMCs were transferred to 96 well plates and incubated with antibodies against extracellular targets (Table 1) on ice for 30 minutes. Cells were fixed and permeabilized using the FoxP3 Transcription Factor Staining Buffer Kit (eBioscience) following the manufacturer’s protocol. Following permeabilization, cells from hu-NSG mice were incubated with antibodies against intracellular targets (Table 1) on ice for 30 minutes. Cells were washed, resuspended in PBS/FBS, and stored at 4°C until analysis. Samples were analyzed on the LSRII Fortessa (BD) using the HTS module. Data were collected using the FACSDiva software (BD). Data were analyzed using FlowJo (v10.2; FlowJo, LLC) (S2 Fig).

**Table 1:** Antibodies used for flow cytometry experiments.

Target	Extracellular (EC), Intracellular (IC), or Nuclear (N)	Panel(s)^a^	Clone	Fluor^b^	Manufacturer
CD1c	EC	2	L161	Pacific Blue	Biolegend
CD3	EC	P1, P2, P3	UCHT1	PE/CF594	BD Biosciences
		1, 2	UCHT1	BUV661	BD Biosciences
CD4	EC	P2, P3, 1	OKT4	BV650	Biolegend
CD8a	EC	P2	RPA-T8	BV570	Biolegend
		1	RPA-T8	BV605	Biolegend
CD11b	EC	P1, P3, 2	ICRF44	BV605	Biolegend
CD11c	EC	P1, P3	Bu15	Pacific Blue	Biolegend
		2	3.9	BV650	Biolegend
CD14	EC	P1, P3, 2	HCD14	AF700	Biolegend
CD16	EC	P1, P3	3G8	BV570	Biolegend
		1	3G8	AF700	Biolegend
CD19	EC	P1, P3, 2	HIB19	PE/Cy7	Biolegend
CD20	EC	P1	2H7	BV650	Biolegend
CD25	EC	P2, 1	BC96	APC/Cy7	Biolegend
CD45	EC	P1, P2, P3, 1, 2	2D1	Amcyan	BD Biosciences
CD56	EC	P1	MEM-188	PerCP/Cy5.5	BD Biosciences
		P2, 1	HCD-56	BV421	Biolegend
CD62L	EC	P2	DREG-56	AF700	Biolegend
CD66b	EC	P1, 2	1A4	PE	BD Biosciences
CD69	EC	1, P2	FN50	PE/Cy5	Biolegend
CD80	EC	2	2D10	APC	BD Biosciences
CD86	EC	2	IT2.2	PE/Cy5	BD Biosciences
CD123	EC	2	7G3	PE/CF594	BD Biosciences
CD177	EC	2	MEM-166	FITC	Biolegend
FoxP3	N	P2, 1	150D	AF647	Biolegend
HLA-DR	EC	P1, 2	L243(G46-6)	APC/Cy7	BD Biosciences
IFNα2b	IC	P3	7N4-1	AF647	BD Biosciences
IFNγ	IC	P3, 1	4S.B4	AF488	Biolegend
IL-2	IC	P3	MQ1-17H12	APC/Cy7	Biolegend
IL-4	IC	P2	MP4-25D2	PE/Cy7	Biolegend
		P2	MP4-25D2	BV711	Biolegend
		1	MP4-25D2	PE/Dazzle 594	Biolegend
IL-8	IC	P3	G265-8	BV510	Biolegend
IL-10	IC	P2, 1	JES3-19F1	PE	Biolegend
IL-12 p40	IC	P3	eBioHP40	PerCP/eFluor 710	eBioscience
IL-17	IC	1	N49-653	PerCP/Cy5.5	Biolegend
Ki67	N	P2	B56	AF488	BD Biosciences
		P2	B56	BV711	BD Biosciences
		2	B56	BV786	BD Biosciences
TGFβ	IC	1	TWF-2F8	PE/Cy7	Biolegend
TNFα	IC	P3	MAb11	BV711	Biolegend
		1	Mab11	BV785	Biolegend
TNFβ	IC	P3	359-81-11	PE	Biolegend

^a^ Panels P1, P2, and P3 all refer to panels used in the preliminary flow studies reported in Figs 3 and 4. Panels 1 and 2 refer to panels used in the remainder of the flow cytometry experiments reported in Figs 5 and 6.

^b^ Some panels contain multiple antibodies to the same target but conjugated to different fluors. These antibodies were not used in conjunction with each other but were substituted out due to manufacturer availability.

### Multiplex cytokine bead array assay

Cytokine and chemokine levels in PBMC supernatant and mouse serum samples were determined using the Milliplex 41-plex human cytokine/chemokine magnetic bead panel (Millipore) according to the manufacturer’s instructions. Samples were collected on either the Bio-Plex (Bio-Rad) using Bio-Plex Manager software or the Magpix (Millipore) using xPonent software (Luminex).

### Statistical analysis

Statistical analysis was performed using Prism (v6.0; GraphPad) software. Outliers were removed using ROUT analysis (Q = 1%). Data were analyzed via two-way ANOVA and t-test using Holm-Sidak correction for multiple comparisons.

### Ethics statement

All experiments involving mice were done in accordance with guidelines of the Institutional Animal Care and Use Committee at Baylor College of Medicine (IACUC Protocol AN-6151), and the recommendations in the *Guide for the Care and Use of Laboratory Animals* (Institute for Laboratory Animal Research, National Research Council, National Academy of Sciences, 2011). The Institutional Review Board at Baylor College of Medicine determined that our use of human blood products in this study did not constitute human subjects research.

## Results

### Mosquito saliva increases the frequency of natural killer T cells in human peripheral blood mononuclear cells

Our previous studies demonstrated that mosquito saliva enhances dengue infection in humanized mice [7]. Therefore, we hypothesized that mosquito saliva modulates the human immune response to enable virus replication. To determine which type of effects we might see in human immune cells, we studied the *ex vivo* responses of primary human peripheral blood mononuclear cells (PBMCs) to mosquito saliva.

**Fig 1:**
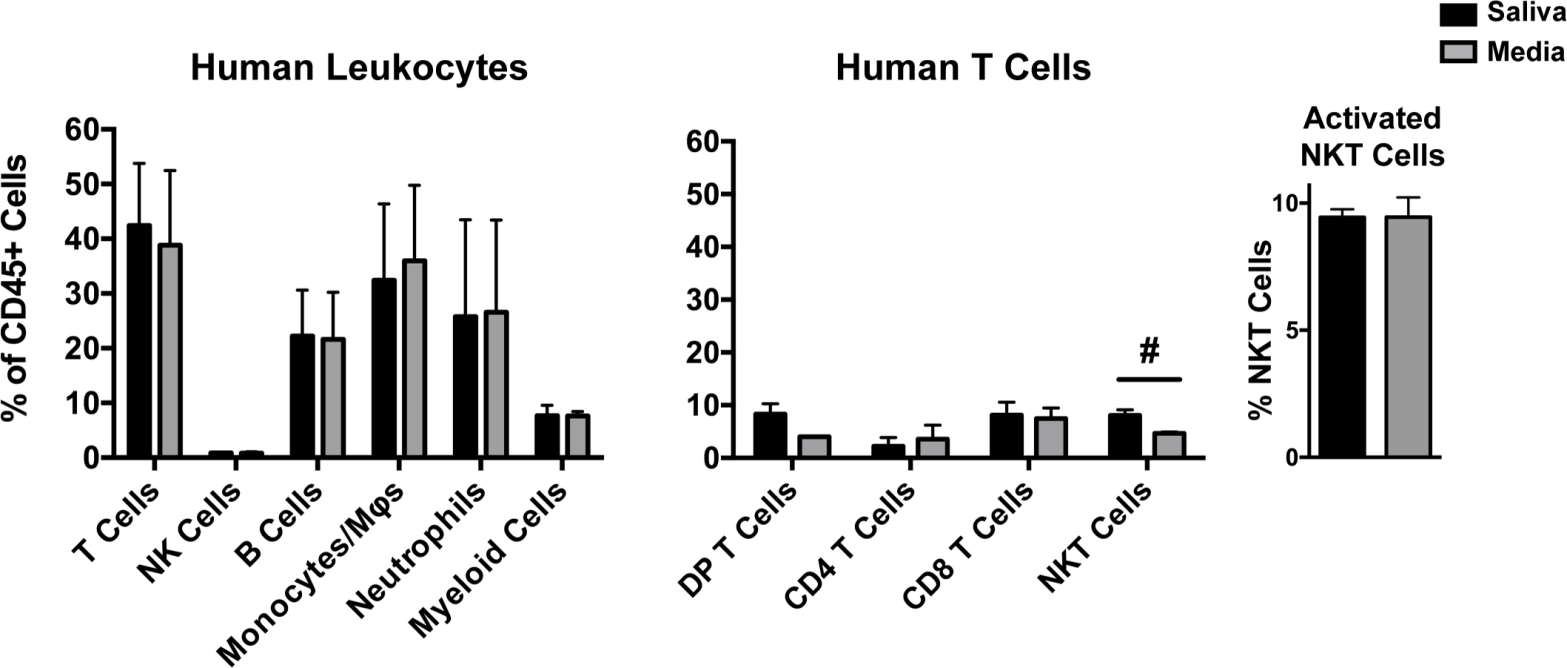
Mosquito saliva increased frequency of CD4+CD8+ double positive T cells and natural killer T cells. Human PBMCs from two donors were stimulated with either mosquito saliva or untreated media. Flow cytometry was performed on these cells 5 days post stimulation to assess frequencies of different immune cell populations. These frequencies are expressed as the mean percentage of CD45+ cells with error bars indicating 1 standard error of the mean (SEM). The subset of the T cell plot represents the percentage of NKT cells that were activated. T-tests were performed using GraphPad Prism. Multiple comparisons were corrected for using the Holm-Sidak method, which increased the significance threshold to p < 0.01. #, p<0.1; ##, p<0.05; *, p<0.01; **p<0.005. Abbreviations: NK = Natural Killer; MΦ = Macrophage; DC = Dendritic Cell; DP = Double Positive. Cell markers used to define cell populations: T cells, CD45+CD3+; NK Cells, CD45+CD3-CD56+; B Cells, CD45+CD3-CD19+; Monocytes/MΦs, CD45+CD3-CD14+; Neutrophils, CD45+CD3-CD66b+; Myeloid Cells, CD45+CD3-CD11c+; DP T Cells, CD45+CD3+CD4+CD8+; CD4 T Cells, CD45+CD3+CD4+CD8-; CD8 T Cells, CD45+CD3+CD4-CD8+; NKT Cells, CD45+CD3+CD56+; Activated NKT Cells, CD45+CD3+CD56+CD69+.

Human PBMCs were isolated from two separate blood donors and then treated with mosquito saliva (equal to 4 mosquito bites: 2.0 μg [37]) or untreated media. We examined the immune cell populations present 5 days post treatment via flow cytometry (Fig 1). Natural killer T cells (NKT; CD3+CD56+) were found at a higher frequency following mosquito saliva treatment; however, we saw no statistical differences in the number of activated cells (CD69+). When activated, NKT cells can rapidly produce large amounts of IFNγ, IL-4, and/or IL-17A. These cytokines can polarize the immune system towards a Th1, Th2, or Th17 response, respectively. [26–28].

### Mosquito saliva suppresses cytokine production in human PBMC cultures

Cytokines are secreted signaling proteins of the innate immune system and are crucial in establishing an effective immune response; thus, we investigated whether mosquito saliva has an effect on cytokines produced by human PBMCs *ex vivo*. Supernatants were collected daily from human PBMCs treated with either mosquito saliva or untreated media; cytokine levels were assessed using multiplex cytokine bead array assays. As positive controls, PBMCs were stimulated with lipopolysaccharide or pokeweed mitogen to verify that these PBMCs were able to produce cytokines (S1 Fig).

**Fig 2:**
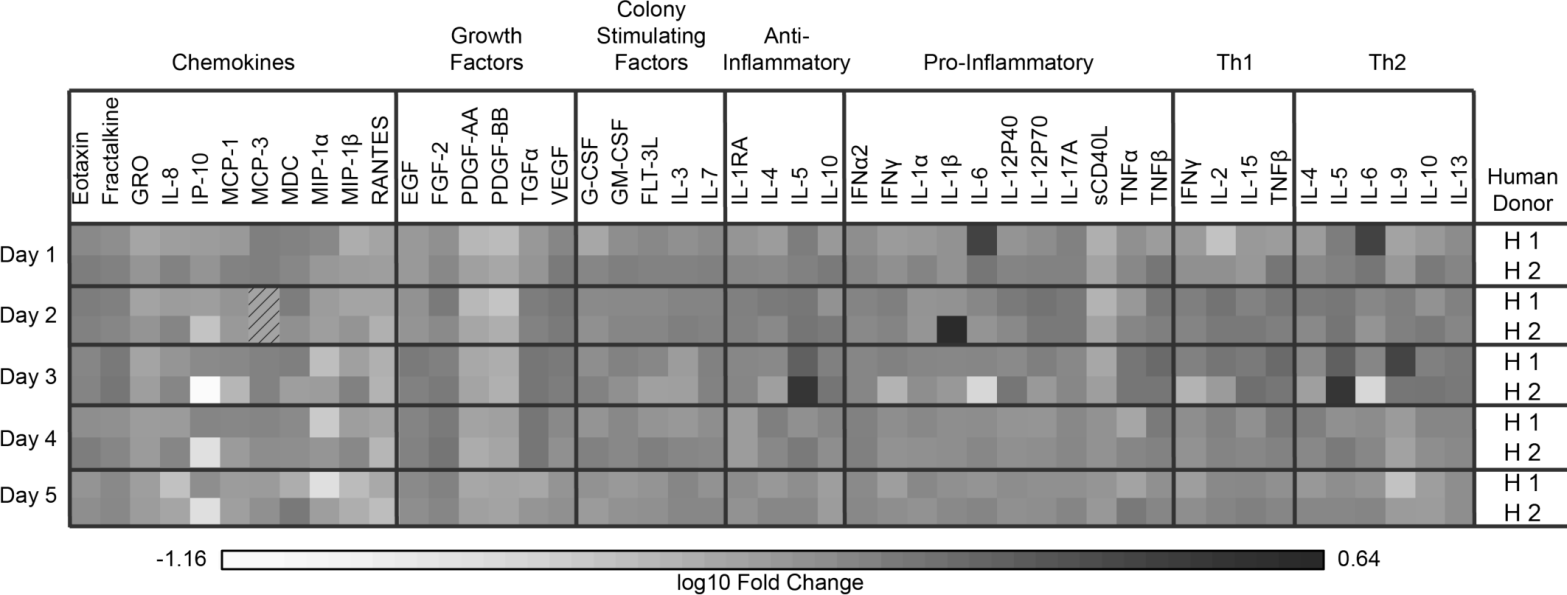
Mosquito saliva decreases cytokine production in human PBMCs. Human PBMCs from two donors were stimulated with either mosquito saliva or untreated media. Supernatant cytokine concentrations were assessed daily via multiplex cytokine bead array assay. Data presented are log10- fold change of saliva stimulated PBMCs compared to media stimulated PBMCs of the same donor; the darker the color, the higher the concentration of the cytokine in the supernatant of the saliva-stimulated PBMCs. Slashed squares represent missing data.

Mosquito saliva-treated PBMCs had lower supernatant concentrations at all time points of most cytokines examined compared to those of the untreated control, with the most significant decreases at days 1 and 2 post treatment (Fig 2). The most significantly lowered cytokines were PDGF-AA, PDGF-BB, and RANTES, all of which are secreted in response to endothelium wall breach and promote clotting and immune cell recruitment [38, 39]. These functions would antagonize mosquito feeding; thus, suppression of these cytokines by mosquito saliva is not unexpected. However, RANTES does regulate CD8+ T cell functions during West Nile virus and HIV infections, so suppression of this cytokine by mosquito saliva could impact the anti-viral immune response [40]. We also observed a decrease in levels of IP-10 in saliva-treated PBMCs; IP-10 is a chemokine that can attract a wide variety of immune cells, including monocytes, macrophages, and dendritic cells [41]. In the context of dengue virus infection, IP-10 has been shown to have a protective effect by competitively inhibiting the access of dengue virus to heparin sulfate, a putative host cell receptor [42, 43]; thus, mosquito saliva could potentially enhance infection by decreasing IP-10 levels. Currently, we are unable to determine whether mosquito saliva actively inhibits the process of cytokine production or merely kills the cells responsible for producing the cytokines.

### Preliminary flow cytometry studies

**Fig 3:**
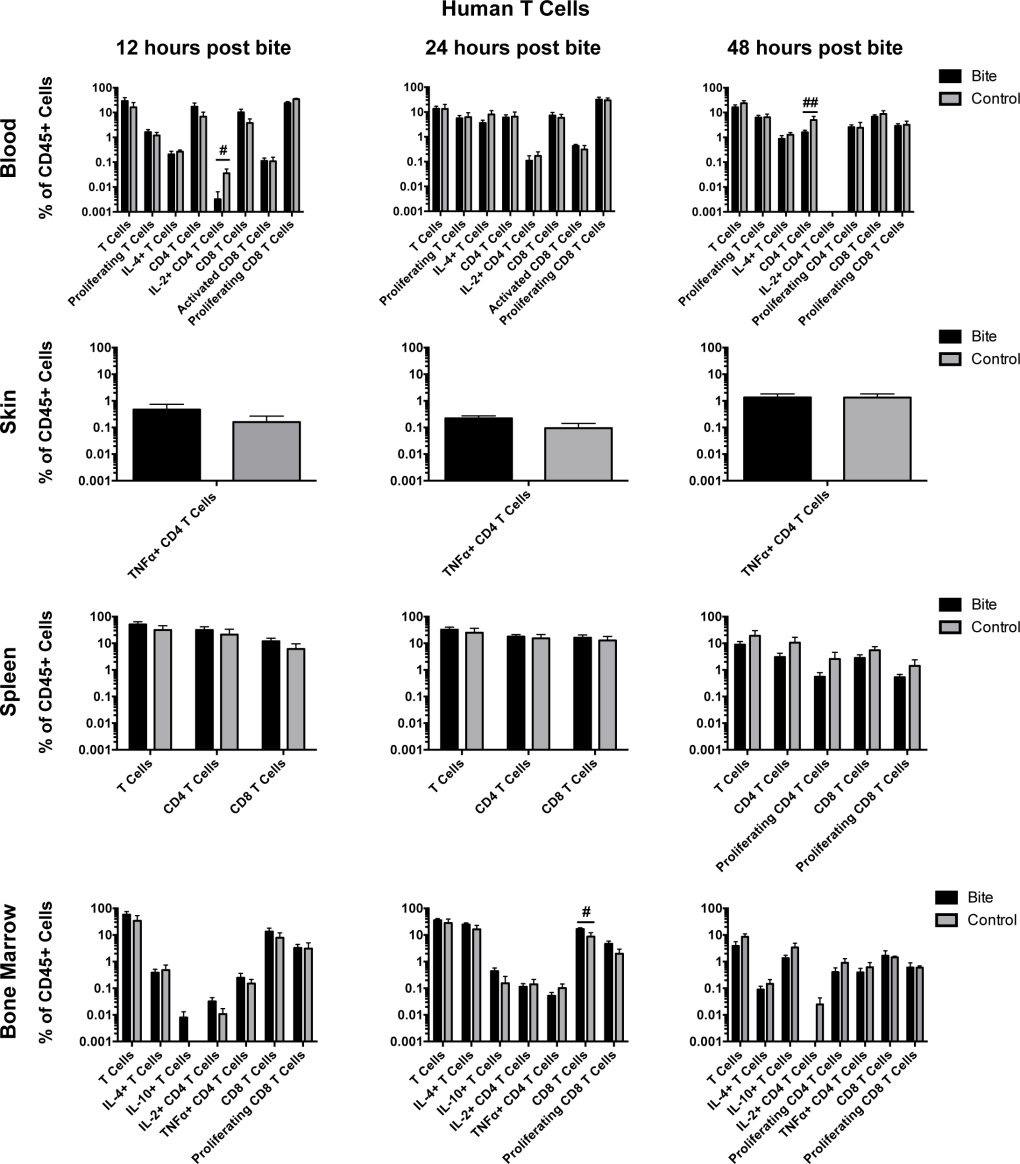
Preliminary assessment of mosquito saliva on human T cell populations in hu-NSG mice. Hu-NSG mice were each bitten on their rear footpads by approximately four mosquitoes. Unbitten mice were used as controls. At 12, 24, or 48 hours post-bite, mice were euthanized and the indicated tissues were collected. Human T cell populations were assessed via flow cytometry. Graphs show the mean frequency of immune cell populations (with outliers removed) as a percentage of human CD45+ cells in bitten (n = 6, 12 or 48 hours post-bite; n = 5, 24 hours post-bite) or control mice (n = 4, 12 or 48 hours post-bite; n = 5, 24 hours post-bite). Error bars represent 1 SEM. T-tests were performed using Graphpad Prism. Multiple comparisons were corrected for using the Holm-Sidak method, which increased the significance threshold to p < 0.01. #, p<0.1; ##, p<0.05; *, p<0.01; **p<0.005. Cell markers used to define cell populations: T Cells, CD45+CD3+; Proliferating T Cells, CD45+CD3+Ki67+; CD4 T Cells, CD45+CD3+CD4+; Proliferating CD4 T Cells, CD45+CD3+CD4+Ki67+; CD8 T cells, CD45+CD3+CD8+; Activated CD8 T Cells, CD45+CD3+CD8+CD69-; Proliferating CD8 T Cells, CD45+CD3+CD8+Ki67+.

**Fig 4:**
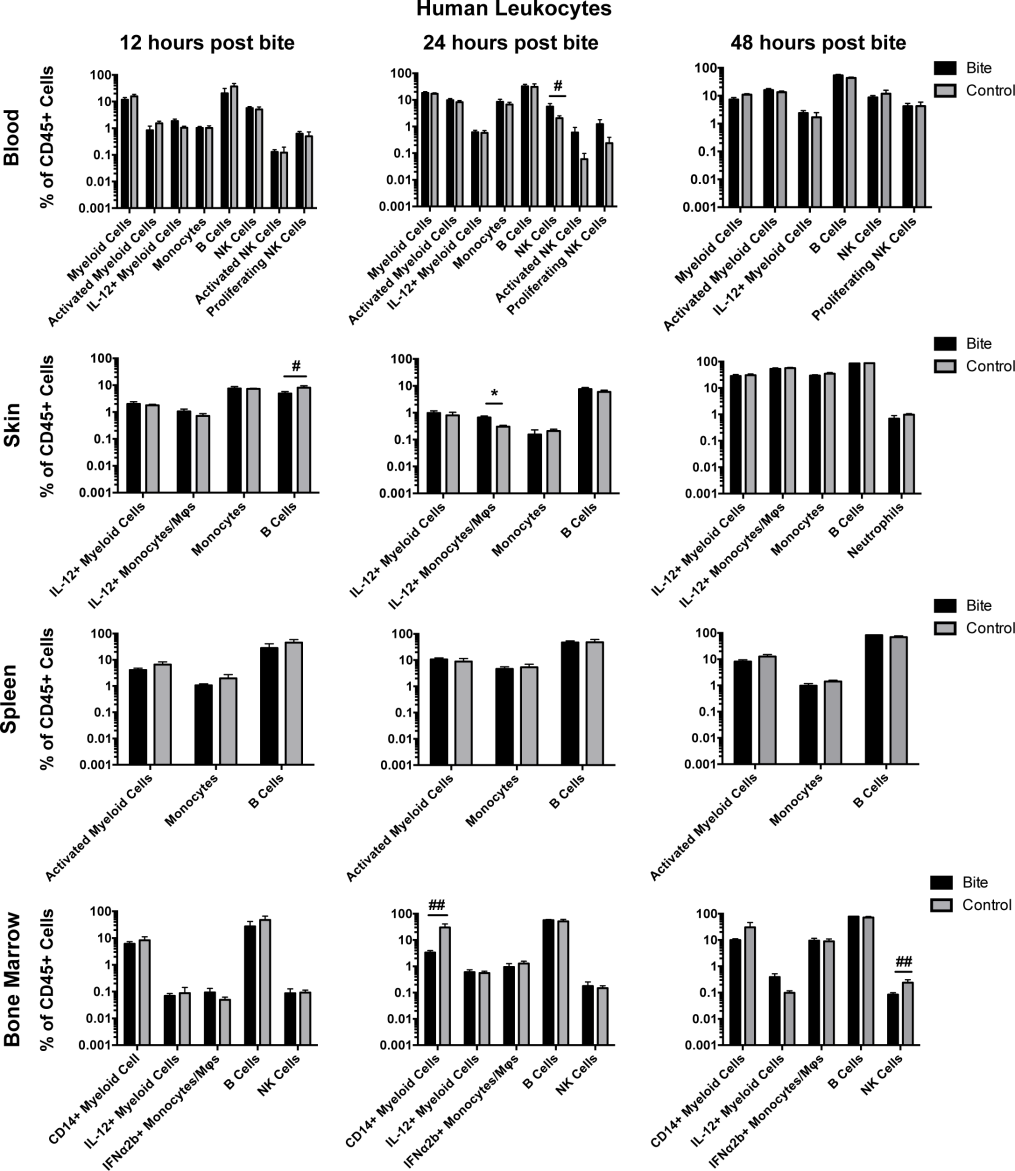
Preliminary assessment of mosquito saliva on human leukocyte populations in hu-NSG mice. Hu-NSG mice were each bitten on their rear footpads by approximately four mosquitoes. Unbitten mice were used as controls. At 12, 24, or 48 hours post-bite, mice were euthanized and the indicated tissues were collected. Human leukocyte populations were assessed via flow cytometry. Graphs show the mean frequency of immune cell populations (with outliers removed) as a percentage of human CD45+ cells in bitten (n = 6, 12 or 48 hours post-bite; n = 5, 24 hours post-bite) or control mice (n = 4, 12 or 48 hours post-bite; n = 5, 24 hours post-bite). Error bars represent 1 SEM. T-tests were performed using Graphpad Prism. Multiple comparisons were corrected for using the Holm-Sidak method, which increased the significance threshold to p < 0.01. #, p<0.1; ##, p<0.05; *, p<0.01; **p<0.005. Abbreviations: DC = Dendritic Cell; MΦ = Macrophage; NK = Natural Killer. Cell Markers used to define cell populations: Myeloid Cells, CD45+CD3-CD11c+; Activated Myeloid Cells, CD45+CD3-CD11c+HLADR+; Monocytes, CD45+,CD3-,CD11c+,CD11b+; B Cells, CD45+CD3-CD19+; NK Cells, CD45+CD3-CD56+; Activated NK Cells, CD45+CD3-CD56+CD69+; Proliferating NK Cells, CD45+CD3-CD56+Ki67+; Monocytes/MΦs, CD45+CD3-CD14+.

After observing alterations in cytokine production and NKT cell frequency in PBMCs treated with mosquito saliva, we expanded our study to investigate the effects of mosquito saliva on the immune system of an entire organism. To this end, we used the hu-NSG mouse model (humanized NOD/SCID/IL2-gamma chain null mice (NSG), generated by infusion of human hematopoietic stem cells into NSG mice), which reproduces human myeloid and human lymphoid cell responses and is susceptible to infection by dengue virus [7, 44–47]. To determine the impact of mosquito bite on the population of various immune cells, donor matched hu-NSG mice were each bitten by an average of 4 mosquitoes on their rear footpads while under ketamine/xylazine anesthesia. Mice were euthanized at 12 hours, 24 hours, or 48 hours post-bite. Skin (rear footpads), blood, spleen, and bone marrow were examined for immune cell population changes to capture both the local and the systemic changes in response to mosquito bite. Unbitten mice served as negative controls. Figs 3 and 4 describe the changes in T cell and other leukocyte populations, respectively.

To summarize our results, at 12 hours post-bite, IL-2 producing CD4 T cells (CD45+, CD3+, CD4+, IL-2+) were decreased in the blood in the exposed compared to control mice. Also at 12 hours post-bite, B cells (CD45+, CD3-, CD19+) cells were decreased in the skin. At 24 hours post-bite, NK cells (CD45+, CD3-, CD56+) were increased in the blood. Also, at 24 hours post-bite, CD8 T cells (CD45+, CD3+, CD8+) were increased in the bone marrow while CD14+ myeloid cells (CD45+, CD3-. CD14+, CD11c+) were decreased in the bone marrow. In the skin at 24 hours post-bite, IL-12 producing monocytes and macrophages (CD45+, CD3-, CD14+, IL-12+) were increased. Lastly, at 48 hours, CD4 T cells (CD45+, CD3+, CD4+) were decreased in the blood, and NK cells (CD45+, CD3-, CD56+) were decreased in the bone marrow. Collectively, these results indicate that mosquito saliva modulates the human immune system represented in our humanized mice, and many significant changes occur in the early hours after mosquito bite. These responses include migration of leukocytes and T cells into the blood, skin and bone marrow; this indicated that further effects could probably be measured downstream, later in time after bite.

### Mosquito bite impacts lymphocyte and myeloid cells across multiple tissues

**Fig 5:**
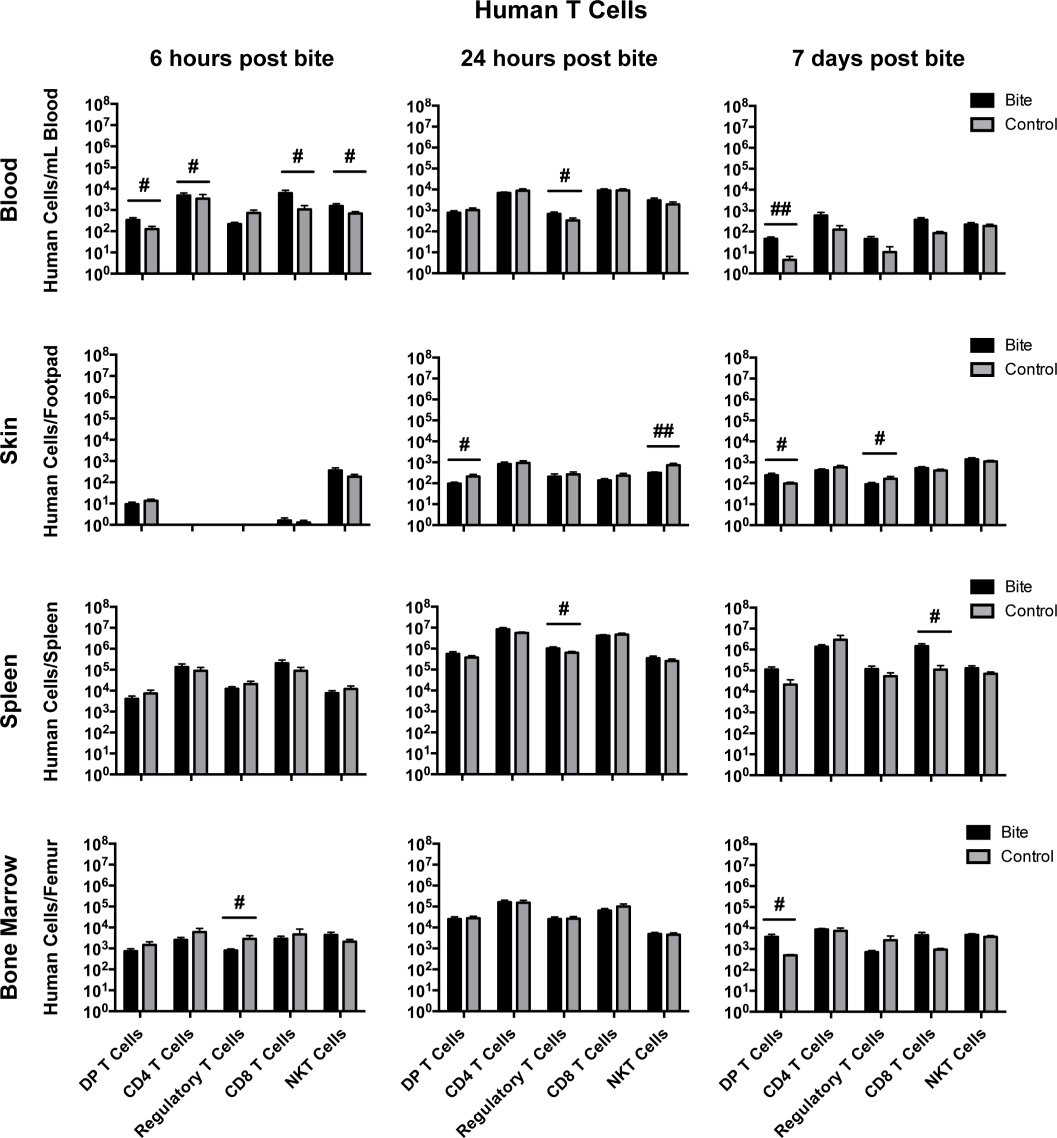
Mosquito saliva changes human T cell populations in hu-NSG mice. Hu-NSG mice were each bitten by approximately four mosquitoes on a rear footpad. Unbitten mice were used as controls. At 6 hours post-bite, 24 hours post-bite, or 7 days post-bite, mice were euthanized and indicated tissues were collected. Immune cell populations were assessed via flow cytometry. Graphs show mean cell counts of bitten (n = 12) and control groups (n = 12) (with outliers removed) as the number of cells per tissue type. Error bars represent 1 SEM. T-tests were performed using GraphPad Prism. Multiple comparisons were corrected for using the Holm-Sidak method, which increased the significance threshold to p < 0.01. #, p<0.1; ##, p<0.05; *, p<0.01; **p<0.005. Abbreviations: DP = Double Positive; NK = Natural Killer. Cell markers used to define cell populations: DP T Cells, CD45+CD3+CD4+CD8+; CD4 T Cells, CD45+CD3+CD4+CD8-; Regulatory T Cells, CD45+CD3+CD4+CD8-FoxP3+; CD8 T Cells, CD45+CD3+CD4-CD8+; NKT Cells, CD45+CD3+CD56+.

**Fig 6:**
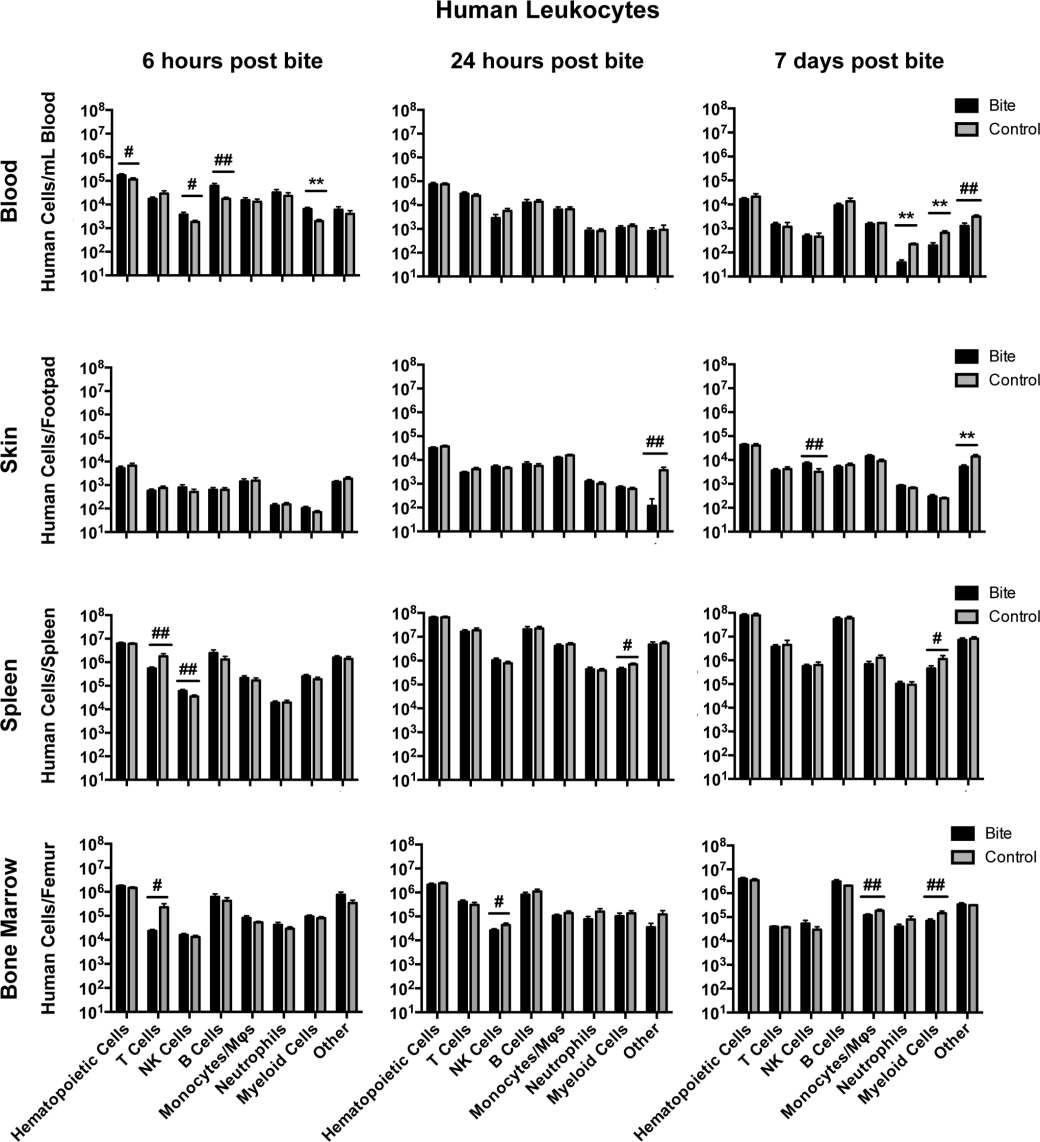
Mosquito saliva alters human leukocyte populations in hu-NSG mice. Hu-NSG mice were each bitten by four mosquitoes on a rear footpad. Unbitten mice were used as controls. At 6 hours post-bite, 24 hours post-bite, or 7 days post-bite, mice were euthanized and indicated tissues were collected. Immune cell populations were assessed via flow cytometry. Graphs show mean cell counts of bitten (n = 12) and control groups (n = 12) (with outliers removed) as the number of cells per tissue type. Error bars represent 1 SEM. T-tests were performed using GraphPad Prism. Multiple comparisons were corrected for using the Holm-Sidak method, which increased the significance threshold to p < 0.01. #, p<0.1; ##, p<0.05; *, p<0.01; **p<0.005. Abbreviations: NK = Natural Killer, Mϕ = Macrophage; DC = Dendritic Cell. Cell markers used to define cell populations: Hematopoietic Cells, CD45+; T Cells, CD45+CD3+; NK Cells, CD45+CD3-CD56+; B Cells, CD45+CD3-CD19+; Monocytes/MΦs, CD45+CD3-CD14+; Neutrophils, CD45+CD3-CD66b+; Myeloid Cells, CD45+CD3-CD11c+; Other, CD45+ but negative for all other markers examined.

Because our preliminary flow cytometry study revealed that mosquito bite impacts the immune system of the hu-NSG mice, we performed a similar experiment using different time points (6 hours, 24 hours, and 7 days post-bite) to deepen our knowledge of the effects of mosquito saliva on the innate (6 hours, 24 hours) and adaptive (7 days) immune responses. For experiments involving these new time points, mice were bitten and euthanized and their tissues collected as in the preliminary flow cytometry study. Figs 5 and 6, along with Tables 2 and 3, describe the changes in T cell and other leukocyte populations, respectively.

**Table 2:** Changes in T cell populations in bitten hu-NSG mice compared to control mice.

	6 hours post-bite	24 hours post-bite	7 days post-bite
**Blood**	↓ Regulatory T Cells ↑ DP T Cells ↑ CD8+ T Cells ↑ NKT Cells	↑ Regulatory T Cells	↑ DP T Cells
**Skin**	No Significant Changes	↓ DP T Cells	↓ Regulatory T Cells ↑ DP T Cells
**Spleen**	↓ Total T Cells	↓ Regulatory T Cells	↑ CD8+ T Cells
**Bone marrow**	↓ Total T Cells ↓ Regulatory T Cells	No Significant Changes	↑ DP T Cells

**Table 3:** Changes in B cells, natural killer cells, and myeloid cells in bitten hu-NSG mice compared to control mice.

	6 hours post-bite	24 hours post-bite	7 days post-bite
**Blood**	↑ B cells ↑ NK Cells ↑ Myeloid Cells	No significant changes	↓ Neutrophils ↓ Myeloid Cells
**Skin**	No Significant Changes	No significant Changes	↑ NK Cells
**Spleen**	↑ Natural Killer Cells	↓ Myeloid Cells	↓ Myeloid Cells
**Bone Marrow**	No Significant Changes	↓ NK Cells	↓ Monocytes/Macrophages ↓ Myeloid Cells

At 6 hours post-bite in the blood, we observed an increase in NKT cells (CD45+, CD3+, CD56+), CD8 T cells (CD45+, CD3+, CD4-, CD8+), and DP T cells (CD45+, CD3+, CD4+, CD8+), which all have cytotoxic capabilities, and a decrease in regulatory T cells (Tregs; CD45+, CD3+, CD4+, FoxP3+), which can dampen inflammatory responses through secretion of IL-10. Also, B cells (CD45+, CD3-, CD19+), NK cells (CD45+, CD3-, CD56+) and myeloid cells (CD45+, CD3-, CD11c+; dendritic cells, monocytes, macrophages, neutrophils) were increased at 6 hours in the blood. The increase of B cells we observed is likely a result of increased production of B cells in the bone marrow. Most myeloid cells are antigen-presenting cells; their increase in the blood may indicate that they are travelling either to or from the bite site. In the spleen, there was an increase in NK cells, but a decrease in the total number of T cells (CD45+, CD3+), and in the bone marrow there were decreases in both total T cells and Tregs. Considering that we observed an increase in multiple T cell subsets in the blood, the reduction of T cells in these two organs could be a result of migration to the blood. Taken together, these results indicate that the immune system is not at steady-state and may be shifting toward a Th1 response; future studies will verify that conclusion by determining which cytokines, if any, are being produced by the DP T, NKT, NK, and CD11c+ cells.

At 24 hours post-bite, Tregs were increased in the blood and decreased in the spleen. The decrease of Tregs in the spleen is coincident with an increase of Tregs in the blood and could indicate that the Tregs migrated from the spleen to the blood. If the increase of Tregs in the blood were associated with an increase in serum IL-10 levels, then this could indicate an anti-inflammatory response. In the skin, DP T cells were decreased at 24 hours post-bite. Because DP T cells are often associated with inflammatory responses, this could indicate a decrease in inflammation in the skin. We also saw a decrease in myeloid cells in the spleen and NK cells in the bone marrow. Taken together, these results indicate a shift towards an anti-inflammatory response.

At 7 days post-bite, DP T cells were increased in the blood, skin, and bone marrow. These DP T cells are typically found at sites of inflammation, indicating that there may be skin inflammation occurring at this time point. There was also a decrease in Tregs and an increase in NK cells in the skin, providing a further indication of inflammation in the skin. We also saw a decrease in neutrophils and myeloid cells in the blood. Because inflammation and DP T cells are associated with both Th1 and Th2 responses, further experiments will need to be done to determine the role of DP T cells following mosquito bite. We saw a decrease in neutrophils and myeloid cells in the blood and a decrease in myeloid cells and monocytes and macrophages cells in the bone marrow. Lastly, there was an increase in CD8 T cells but a decrease in myeloid cells in the spleen. CD8 T cells are typically associated with a Th1 response and can produce large amounts of pro-inflammatory cytokines. Further experiments will determine whether mosquito saliva activates these CD8 T cells and stimulates them to produce cytokines. While T cells and NK cells indicate an inflammatory response, the overall data suggest a mixed Th1/Th2 response.

### Mosquito bite alters cytokine levels in hu-NSG mice

**Fig 7:**
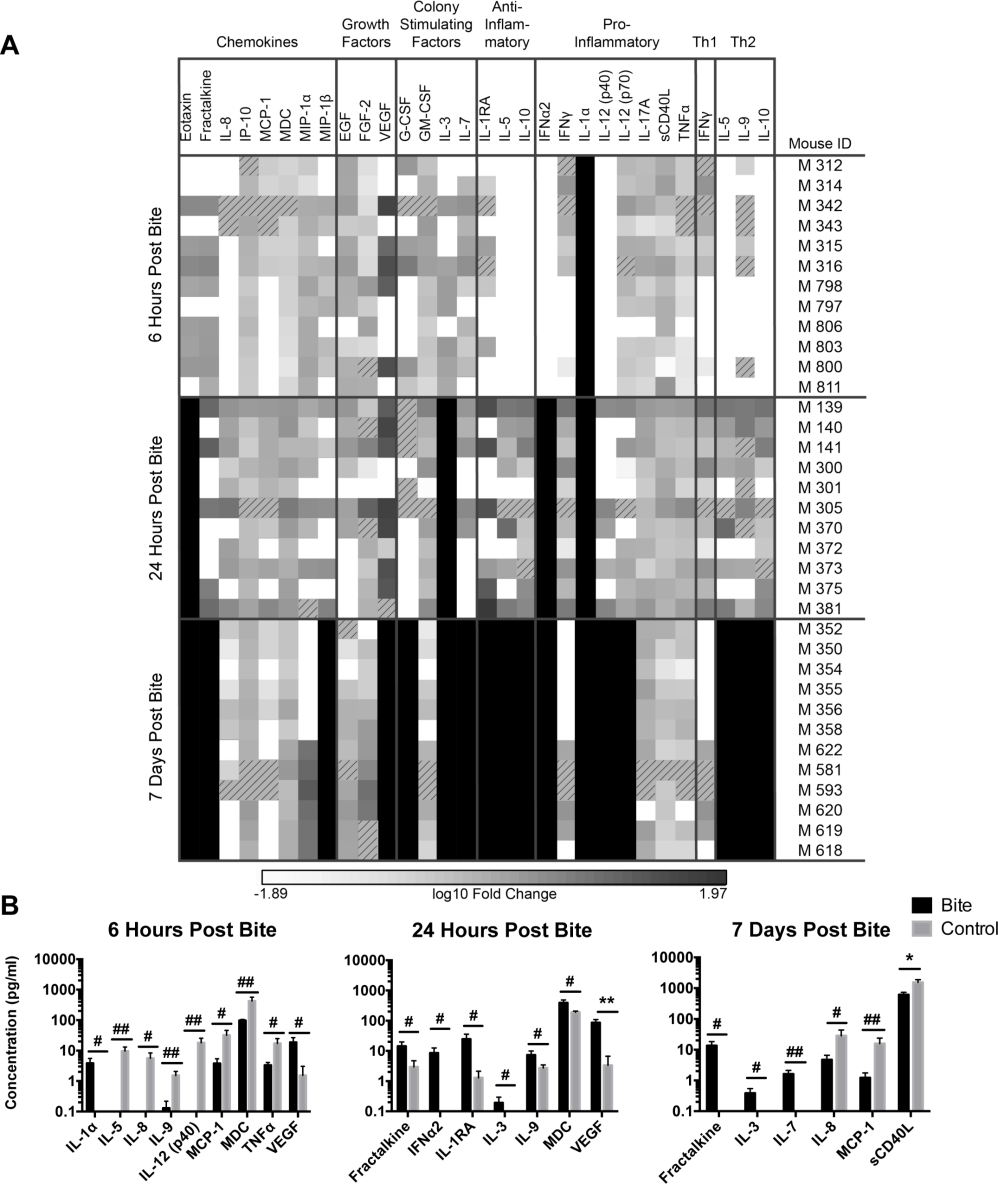
Mosquito saliva alters concentrations of select serum cytokines in hu-NSG mice. (A) Serum cytokine production in hu-NSG mice following mosquito bite. Hu-NSG mice were each bitten by approximately four mosquitoes on a rear footpad. Unbitten mice were used as controls. At the indicated times, mice were euthanized and serum cytokine levels were determined via multiplex cytokine bead array assay. Data presented are the log_10_ fold change of specific cytokine concentrations in each bitten mouse compared to the mean concentration of the control mice. Darker colors represent a higher concentration of a given cytokine in the serum of the bitten mice. Pure black represents a positive ∞ log_10_ fold change (highest possible), while pure white represents a negative ∞ log_10_ fold change (lowest possible). Slashed squares represent missing data or outlier data. Data for the following cytokines were not included in the analysis because all mice had serum concentrations below the limit of detection: Flt3 ligand, GRO, IL-1β, IL-2, IL-4, IL-6, IL-13, IL-15, MCP3, TGFα, and TNFβ. (B) Significant differences in serum cytokine levels between bitten mice (black) and control mice (gray). Graphs show mean concentrations (pg/mL) of cytokines; error bars are 1 SEM. T-tests were performed on serum cytokine concentration data (with outliers removed) from (A) using GraphPad Prism. Multiple comparisons were corrected for using the Holm-Sidak method, which increased the significance threshold to p < 0.01. #, p<0.1; ##, p<0.05; *, p<0.01; **p<0.005.

To further our examination of mosquito bites on the immune system of humanized mice, we investigated whether serum levels of human cytokines changed in mosquito-bitten mice. Serum was collected at 6 hours, 24 hours, and 7 days post-bite from the same mice used to examine immune cell populations, and cytokine levels were measured via multiplex cytokine bead array assays. Fig 7A shows the fold-change of each bitten mouse compared to the mean of the control group. Cytokines were grouped by the following functions to assist in analysis: chemokines, growth factors pro-inflammatory cytokines, anti-inflammatory cytokines, Th1 cytokines, and Th2 cytokines. A majority of the bitten mice did not appear to differ from the control in cytokine levels; however, the fold change differences in Th2 cytokines and anti-inflammatory cytokines increased over time.

Despite a lack of significant changes across broad classes of cytokines, we did see significant changes in concentrations of specific cytokines. At 6 hours post-bite, we saw an increase in the pro-inflammatory cytokine IL-1α, but a decrease in the pro-inflammatory cytokines IL-12p40 and TNFα. In addition to promoting inflammation, IL-12p40 stimulates the production of IFNγ (the major Th1 cytokine) by NK cells and T cells [48]. We also saw a decrease in the Th2 cytokines IL-5 and IL-9 at 6 hours post- bite. Both of these cytokines are produced by T cells [49, 50]; from our examination of T cell populations in the hu-NSG mice following mosquito bite (Fig 5), we saw a significant decrease in total T cells in the spleen and bone marrow and a trend toward a decrease in total T cells in the skin and blood at 6 hours post-bite. This decrease in T cells may account for the decreased serum concentrations of IL-5 and IL-9. We also saw a decreased serum concentration of the chemokines IL-8, MCP-1, and MDC at this time point. Lastly, we saw an increase in the growth factor VEGF at this time point; VEGF is often released in response to endothelium wall breach to stimulate the growth of endothelial cells [51, 52]. Since the mosquito bites likely caused limited vascular injury in the mice, it is not unexpected that we observed an increase in serum VEGF in the bitten mice compared to the control mice. To summarize, the serum cytokine concentrations at 6 hours post- bite indicate a dysregulation of inflammatory signaling, a decrease in Th1 and Th2 signaling, an alteration in chemotactic signaling, and an increase in endothelium repair signaling.

At 24 hours post-bite, we saw an increase in the pro-inflammatory and anti-viral cytokine IFNα [53]. This cytokine is usually produced in response to viral infection, so its presence after uninfected mosquito bite is unexpected. To our knowledge, this is the first report of IFNα production following mosquito bite. We saw a decrease in the anti-inflammatory cytokine IL-1RA, a cytokine that competitively inhibits IL-1α and IL-1β from binding to the IL-1 receptor [54, 55]. The increase in this cytokine may be an attempt to regulate the increased IL-1α observed at 6 hours post-bite. We saw an increase in the Th2 cytokine IL-9; this increase contrasts with our observation at 6 hours post-bite where a decrease in IL-9 was coincident with a decrease in the number of total T cells. At 24 hours post-bite, numbers of total T cells in the bitten mice is equivalent to that of the control mice, which may account for the increase in serum concentrations of IL-9. We saw an increase in the chemokines fractalkine and MDC. An increase in these chemokines in the serum could induce an influx monocytes and other cells that express the appropriate chemokine receptors into the blood [56–58]. We did not see any shifts in monocyte populations in the mice at 24 hours post-bite, but perhaps this shift occurred at a time point that was not analyzed. Lastly, we saw an increase in the growth factors VEGF and IL-3. As previously mentioned, VEGF is involved in endothelium wall repair; IL-3 is also involved in endothelium wall repair and promotes the production of platelets, which are consumed during an endothelium wall breach [59]. To summarize, the serum cytokines present at 24 hours post-bite indicate an interferon response, an anti-inflammatory response, a Th2 response, an influx of monocytes, and a continued endothelium repair response.

At 7 days post-bite, we saw a decrease in the pro-inflammatory cytokine sCD40L in the bitten mice compared to the control mice. In addition to stimulating an inflammatory response, this cytokine is also important for immunoglobulin class switching by B cells [60–62]. In the context of a viral infection, decreasing or preventing immunoglobulin class switching could be detrimental for the host. We saw an increase in serum concentrations of the chemokine fractalkine and a decrease in the chemokines IL-8 and MCP-1. Since both fractalkine and MCP-1 attract similar cell types (namely monocytes), it is difficult to predict whether changes in concentrations of these two chemokines affect efflux or influx of cells into the blood [56, 57, 63]. While we did not see a significant change in monocyte numbers in the blood, we did see a decrease of monocytes in the bone marrow at this time point. Also, the decrease in IL-8 in the serum did correspond with a decrease in neutrophils (the target of IL-8) in the blood and a trend toward an increase in neutrophils in the skin [64]. We also observed an increase in the growth factors IL-3 and IL-7. IL-7 enhances the growth of early B and T cells and stimulates the proliferation of mature T cells [65, 66]. This increase in IL-7 correlated with an increase in CD8 T cells in the spleen and DP T cells in the blood, skin, and bone marrow. To summarize, at 7 days post-bite, we saw a decrease in pro-inflammatory cytokines, an increase in some but a decrease in other chemokines, and an increase in growth factors.

We were unable to detect the following cytokines in the serum of either the bitten or the control mice at any of the time points: Flt-3 ligand, GRO, IL-1β, IL-2, IL-4, IL-6, IL-13, IL-15, MCP3, TGFα and TNFβ. Of these, GRO, IL-2, IL-4, IL-6, IL-13 and TGFα can be detected in the serum of healthy humans [67–70]. T cells are the primary producers of IL-2, IL-4, IL-6, and IL-13 [71–78]. Because hu-NSG mice do not have a human thymus, the T cells present in these mice are not HLA restricted to human cells and may not respond appropriately to signals from other human immune cells in the mice. This could account for the lack of these cytokines in the serum. Both GRO and TGFα are produced by keratinocytes and monocytes in the skin [79, 80]. Since hu-NSG mice do not have human keratinocytes, these mice would produce low to no levels of GRO and TGFα. Lastly, since we only investigated cytokines present in the serum of these mice, our data do not exclude the possibility that these cytokines were produced in the tissues. Future studies will investigate what cytokines are produced at the site of mosquito bite and at other tissues throughout the body.

## Discussion

Mosquitoes deposit numerous salivary proteins into their host's skin while acquiring a blood meal. Past work has shown that mosquito saliva enhances the pathogenicity of dengue, West Nile, and other arbovirus infections. From these results, we hypothesized that mosquito saliva modulates the host immune system in a way that promotes arbovirus replication and transmission. In this work, we used human PBMCs treated with mosquito saliva and humanized mice bitten by mosquitoes to determine what effects mosquito saliva alone has on the human immune system. Our results suggest that the human immune response to mosquito saliva is significant and complex: mosquito saliva alters the frequencies of several immune cell populations, in multiple tissues, at several times after blood feeding. Mosquito saliva also affects serum cytokine levels, with the most notable trend being an increase in anti-inflammatory and Th2 cytokines compared to unbitten, control mice, at 7 days post- bite. The fact that these effects last up to 7 days post-bite is especially interesting, and is of concern in the context of allergic reactions. The long-lasting effects in the humanized bone marrow and skin cells could explain how some of the viruses transmitted by mosquitoes could possibly still be viable in these tissues, or how they could serve as replication reservoirs.

Relatively few studies have investigated the effects of mosquito saliva on human cells *ex vivo*; fewer have investigated cytokine production from human cells following stimulation with mosquito saliva. Salivary gland extracts from the *Armigeres subalbatus* mosquito (a vector of Japanese encephalitis and West Nile viruses) induce apoptosis in human PBMCs and THP-1 cells (human monocytic cell line) [81]. In another *ex vivo* study, monocyte-derived dendritic cells differentiated from human PBMCs produced higher amounts of the cytokine IL-12p70 when stimulated with mosquito saliva collected from *Ae*. *aegypti* mosquitoes [82]. In a third study, mosquito saliva from *Anopheles stephensi* mosquitoes (a vector of malaria) induced the degranulation of the human mast cell line LAD-2 [83]. We did not detect a significant decrease in cell populations or an increase in IL-12p70 production in our *ex vivo* studies; however, this is most likely due to differences in study design. We used mosquito saliva as opposed to salivary gland extract (a heterogeneous mixture of saliva and the cells that make it) and monocyte-derived dendritic cells and mast cells are rarely found in the blood and therefore would not be a component of our human PBMCs.

Other mouse studies of the effects of mosquito saliva on the immune system concluded that mosquito saliva primes the immune response toward a Th2 response (reviewed in [84]); however, our results indicate a mixed Th1/Th2 response. We observed increases in both regulatory T cells and CD4/CD8 double positive T cells. As these cells are rare, and more potent cytokine producers and killers than conventional single positive T cells [18–22], it is interesting to speculate that mosquito saliva may be uniquely capable to induce the differentiation of single positive T cells into this cytotoxic double positive T cell subset. Further studies are needed to examine whether these unique double positive T cells play an important role in host protection from flavivirus infection. Furthermore, we noticed decreases in total T cells and natural killer cells after mosquito bite; these decreases may point toward a Th2 response. When analyzing the serum cytokine response, we did observe a significant trend toward Th2 cytokine production at 7 days post-bite. Th2 immune responses occur most often in response to parasitic infections or allergen exposure, and they tend to dampen inflammatory and cytotoxic responses, both of which are needed to clear viral infections. Thus, if mosquito saliva were to trigger a Th2 response in the context of an arbovirus infection, the virus would be able to replicate to higher levels than it would in the presence of a Th1 response. In most arbovirus infections, higher viral load corresponds to more severe disease. Thus, a saliva-induced Th2 immune response could explain observations in experimental models of arbovirus infections that mosquito saliva enhances pathogenesis.

At various times post mosquito bite, we also observed increases in cell populations typically associated with a Th1 immune response: natural killer T cells, natural killer cells, and CD8+ T cells. While these cells can kill virus-infected cells and are generally important for clearing viral infections, they have been implicated in increased disease severity of dengue infection in humans [85]. In cases of dengue hemorrhagic fever in human children, increased numbers of CD69+ natural killer cells, CD4+ T cells, and CD8+ T cells have been observed [86–88]. Some speculate that the increase in these cell populations is a response to severe infection, not a mechanism of pathogenesis; however CD8+ T cells isolated from these severely infected individuals were primed towards producing large amounts of pro-inflammatory cytokines and not towards killing viral infected cells [88, 89]. Further investigations will need to be done to determine whether the increased CD8+ T cell populations we observed were pro-inflammatory CD8+ T cells. Nevertheless, the possibility remains that even though mosquito saliva increases some subsets of immune cells typically associated with a Th1 immune response, these cells could be responsible for increased disease severity in humans.

Our study is not the only one to show that mosquito saliva produces a mixed Th1/Th2 response. Pingen et al discovered that while a mosquito bite does induce some elements of a Th2 response, it did not prevent elements of a Th1 response in C57BL/6 mice [90]. They concluded that mosquito saliva enhances disease by recruiting arbovirus-susceptible cells to the bite site, in their model of Semliki Forest virus infection. Zeidner et al. reached the same conclusion, in "flavivirus-susceptible" (C3H) mice bitten by Aedes mosquitoes, with some T cells and cytokines detectable in the serum up to 7 days post-bite; however, this study did not include flow cytometry, for identification of cell subsets, and these mice are not good models of human infection [34]. In our study, we observed an increase in monocytes and macrophages to the bite site, but only after 7 days post-bite. In the context of arbovirus infection, the migration we observed would be too late to impact the initial replication or dissemination of the virus but could allow for infected cells to return to the skin where they could transmit the virus to new mosquitoes. This is a significant observation that could possibly explain epidemiologic events, where arbovirus transmission is often limited to distinct, small areas of hypertransmission, and where non-viremic hosts could be sources of infection [91–93].

All of the mouse studies of mosquito saliva effects reported previously have used mice that differ from human immune systems: AG129 (which lack type I and type II interferon receptors), BALB/c mice (which are predisposed to Th2 immune responses), and C57BL/6 mice (which are predisposed to Th1 immune responses) [32, 33, 90, 94]. The mouse model we used in this study does not have the same limitations because these mice have been reconstituted with a human immune system, including innate immunity [95]. However, this model does not have either a full human complement system or a fully functional T cell compartment [95]. In future studies, we seek to address these limitations by using other types of reconstituted humanized mice (e.g., BLT, DRAG), which would reproduce different parts of the human immune system (such as active T helper cells, immunoglobulin class-switching, etc.) following mosquito bite [44, 47, 96]. In addition, we expect to test for the biological significance of these immune cell changes, which might lead to stimulation of infected cells to migrate to important sanctuary tissues (e.g., bone marrow or brain), where viral reservoirs could be established, away from the full forces of the immune system. In the case of arboviruses, many establish infections in brain or bone marrow cells of human patients, leading to specific pathologies such as encephalitis or bone loss and leukopenia and thrombocytopenia [97–99]. It is currently unclear if mosquito saliva contributes to these tissue infections and more severe pathologies.

Mosquitoes and the diseases they transmit are of growing public health concern. Often, there are no prophylaxes for these diseases other than mosquito control and no treatments other than palliative care. Understanding how mosquito saliva interacts with the human immune system not only helps us understand mechanisms of disease pathogenesis but also could provide possibilities for treatments. If we know which mosquito saliva components enhance pathogenesis of diseases, we could create a human vaccine to counteract these effects for multiple arbovirus infections. A similar approach has been used to vaccinate and protect mice against a sandfly saliva protein (maxadilan) that enhances the infection and progression of Leishmania major [100]. These approaches have been commercialized and used to interrupt tick transmission of cattle diseases [101], and we expect that the definition of these factors would help provide the same approaches in humans.

## Supporting information

S1 Fig Stimulation with pokeweed mitogen or LPS alters immune cell population frequencies and cytokine production in human PBMCs. Human PBMCs were isolated from two different human donors and stimulated with mosquito saliva, pokeweed mitogen, LPS, or untreated media. Pokeweed mitogen and LPS were used to confirm that the human PBMCs used in these experiments were capable of responding to immunomodulatory compounds. (A) Immune cell population changes following stimulation with pokeweed mitogen and LPS. Stimulated human PBMCs were analyzed via flow cytometry five days post stimulation. Data presented are the mean frequencies of individual cell populations as a percentage of CD45+ cells. The subset of the T cell plot represents the percentage of NKT cells that were activated. Error bars represent 1 standard error of the mean. Abbreviations: NK = Natural Killer; MΦ = Macrophage; DC = Dendritic Cell; DP = Double Positive. Cell markers used to define cell populations: T cells, CD45+CD3+; NK Cells, CD45+CD3-CD56+; B Cells, CD45+CD3-CD19+; Monocytes/MΦs, CD45+CD3-CD14+; Neutrophils, CD45+CD3-CD66b+; DCs/Monocytes/MΦs, CD45+CD3-CD11c+; DP T Cells, CD45+CD3+CD4+CD8+; CD4 T Cells, CD45+CD3+CD4+CD8-; CD8 T Cells, CD45+CD3+CD4-CD8+; NKT Cells, CD45+CD3+CD56+; Activated NKT Cells, CD45+CD3+CD56+CD69+. (B) Cytokine production by human PBMCs following stimulation with pokeweed mitogen and LPS. Supernatants from stimulated human PBMCs were collected daily up to five days post stimulation and analyzed for cytokine concentrations. Data shown are the log10 fold change of each of the stimulated conditions compared to the media stimulated control for the each donor and time point; the darker the color, the higher production of cytokines in treated PBMCs (saliva, pokeweed mitogen, or LPS). Areas with slashes represent samples where no data were collected for that cytokine.
(TIF)

S2 Fig Gating strategy for flow cytometry experiments. These flow charts describe the gating strategies used to analyze flow cytometry data in this study. (A) This was the gating strategy used for the data presented in Figs 3 and 4. Grey boxes represent gates that were made during the analysis of all three panels. Blue, red, and yellow boxes represent gates that were made during the analysis of Panels P1, P2, and P3, respectively. Green boxes represent gates that were made during the analysis of both Panels P1 and P3, and orange boxes represent gates that were made during the analysis of both Panels P2 and P3. (B) This was the gating strategy used for the data presented in Figs 1, 5 and 6. Grey boxes represent gates that were made during the analysis of both Panel 1 and Panel 2 data. Red boxes represent gates that were made only during the analysis of Panel 1 data. Blue boxes represent gates that were made only during the analysis of Panel 2 data. Boxes containing italicized text represent gates that were only used in the analysis of humanized mice samples and not in the analysis of human PBMC samples.
(TIF)

S3 Fig Stereomicroscope photographs of a mouse footpad immediately after 3 mosquito bites, and the mosquitoes that bit that humanized mouse (3 per footpad). There is no evidence of injury or bleeding into the tissues after 3 mosquito bites on each footpad.
(TIF)

S1 Table List of humanized mice used in these experiments. Mice are listed according to experimental group, with mouse ID, sex, and human CD45+ engraftment levels given.
(DOCX)
