# Supplementary figures and images for: Mosquito saliva alone has profound effects on the human immune system
Source: PLoS Negl Trop Dis. 2018 May 17;12(5):e0006439. doi: 10.1371/journal.pntd.0006439 (PMC5957326; doi:10.1371/journal.pntd.0006439)

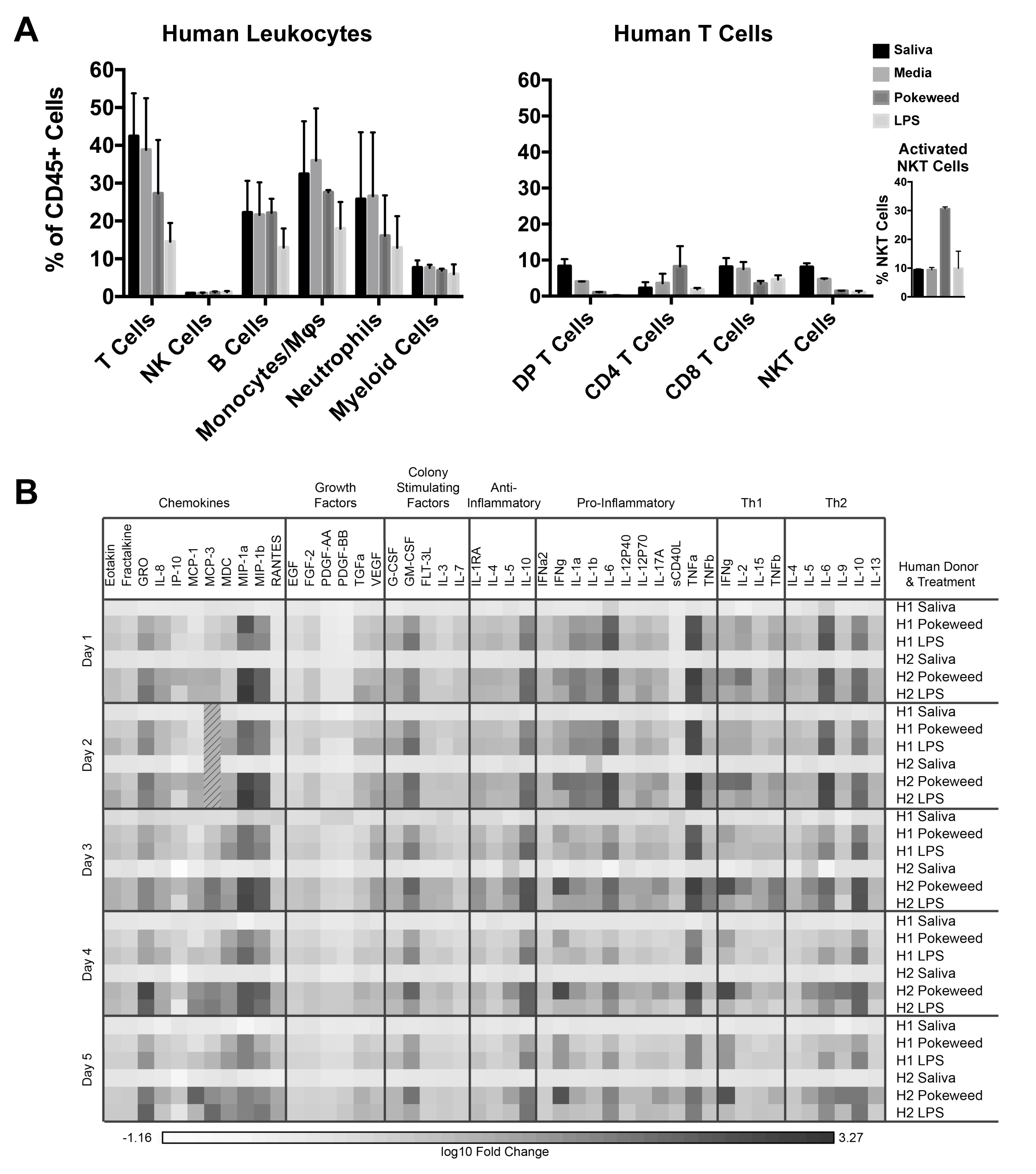

Supplement: S1 Fig — Human PBMCs were isolated from two different human donors and stimulated with mosquito saliva, pokeweed mitogen, LPS, or untreated media. Pokeweed mitogen and LPS were used to confirm that the human PBMCs used in these experiments were capable of responding to immunomodulatory compounds. (A) Immune cell population changes following stimulation with pokeweed mitogen and LPS. Stimulated human PBMCs were analyzed via flow cytometry five days post stimulation. Data presented are the mean frequencies of individual cell populations as a percentage of CD45+ cells. The subset of the T cell plot represents the percentage of NKT cells that were activated. Error bars represent 1 standard error of the mean. Abbreviations: NK = Natural Killer; MΦ = Macrophage; DC = Dendritic Cell; DP = Double Positive. Cell markers used to define cell populations: T cells, CD45+CD3+; NK Cells, CD45+CD3-CD56+; B Cells, CD45+CD3-CD19+; Monocytes/MΦs, CD45+CD3-CD14+; Neutrophils, CD45+CD3-CD66b+; DCs/Monocytes/MΦs, CD45+CD3-CD11c+; DP T Cells, CD45+CD3+CD4+CD8+; CD4 T Cells, CD45+CD3+CD4+CD8-; CD8 T Cells, CD45+CD3+CD4-CD8+; NKT Cells, CD45+CD3+CD56+; Activated NKT Cells, CD45+CD3+CD56+CD69+. (B) Cytokine production by human PBMCs following stimulation with pokeweed mitogen and LPS. Supernatants from stimulated human PBMCs were collected daily up to five days post stimulation and analyzed for cytokine concentrations. Data shown are the log10 fold change of each of the stimulated conditions compared to the media stimulated control for the each donor and time point; the darker the color, the higher production of cytokines in treated PBMCs (saliva, pokeweed mitogen, or LPS). Areas with slashes represent samples where no data were collected for that cytokine. (TIF) [file pntd.0006439.s001.tif]

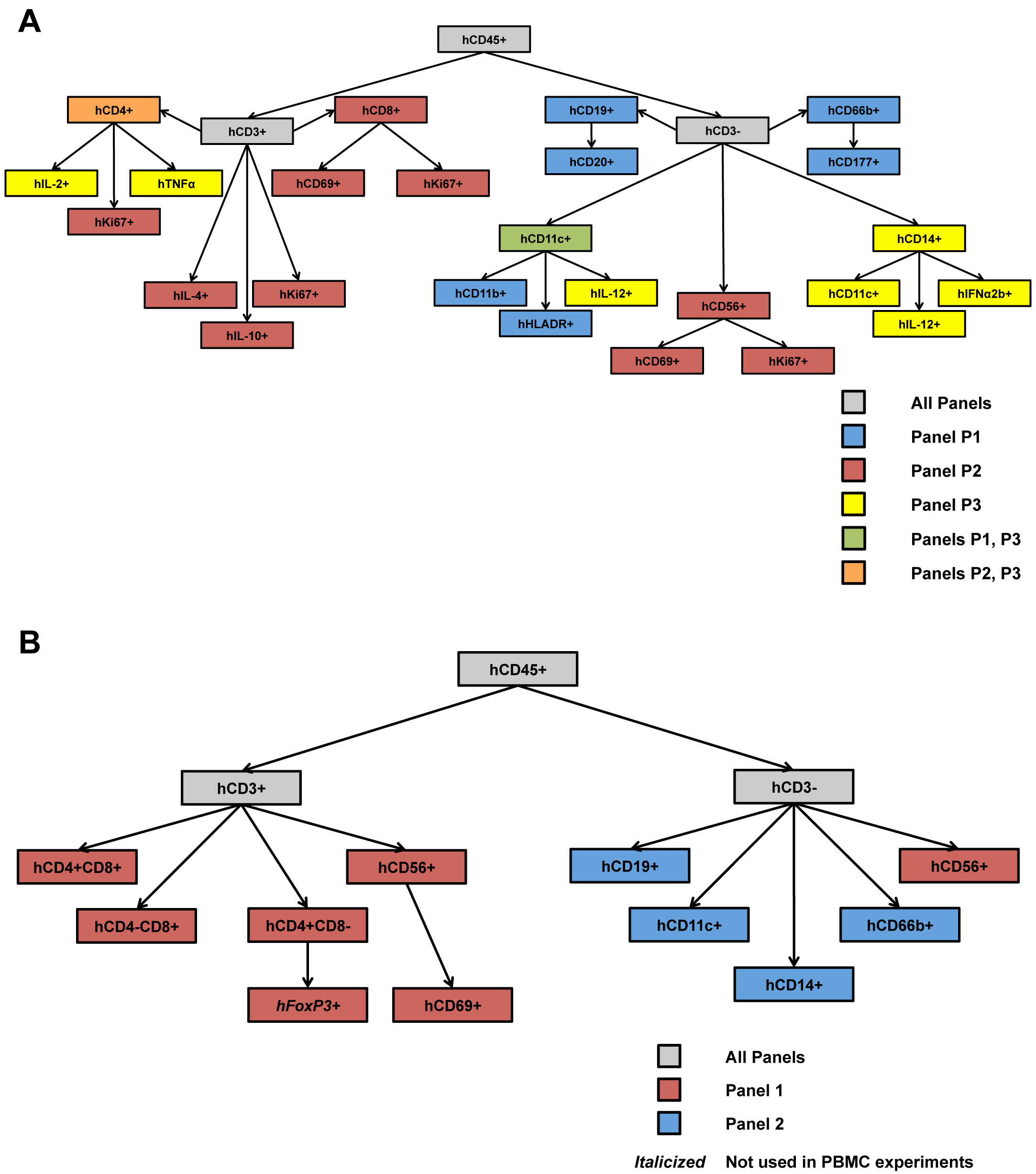

Supplement: S2 Fig — These flow charts describe the gating strategies used to analyze flow cytometry data in this study. (A) This was the gating strategy used for the data presented in Figs 3 and 4. Grey boxes represent gates that were made during the analysis of all three panels. Blue, red, and yellow boxes represent gates that were made during the analysis of Panels P1, P2, and P3, respectively. Green boxes represent gates that were made during the analysis of both Panels P1 and P3, and orange boxes represent gates that were made during the analysis of both Panels P2 and P3. (B) This was the gating strategy used for the data presented in Figs 1, 5 and 6. Grey boxes represent gates that were made during the analysis of both Panel 1 and Panel 2 data. Red boxes represent gates that were made only during the analysis of Panel 1 data. Blue boxes represent gates that were made only during the analysis of Panel 2 data. Boxes containing italicized text represent gates that were only used in the analysis of humanized mice samples and not in the analysis of human PBMC samples. (TIF) [file pntd.0006439.s002.tif]

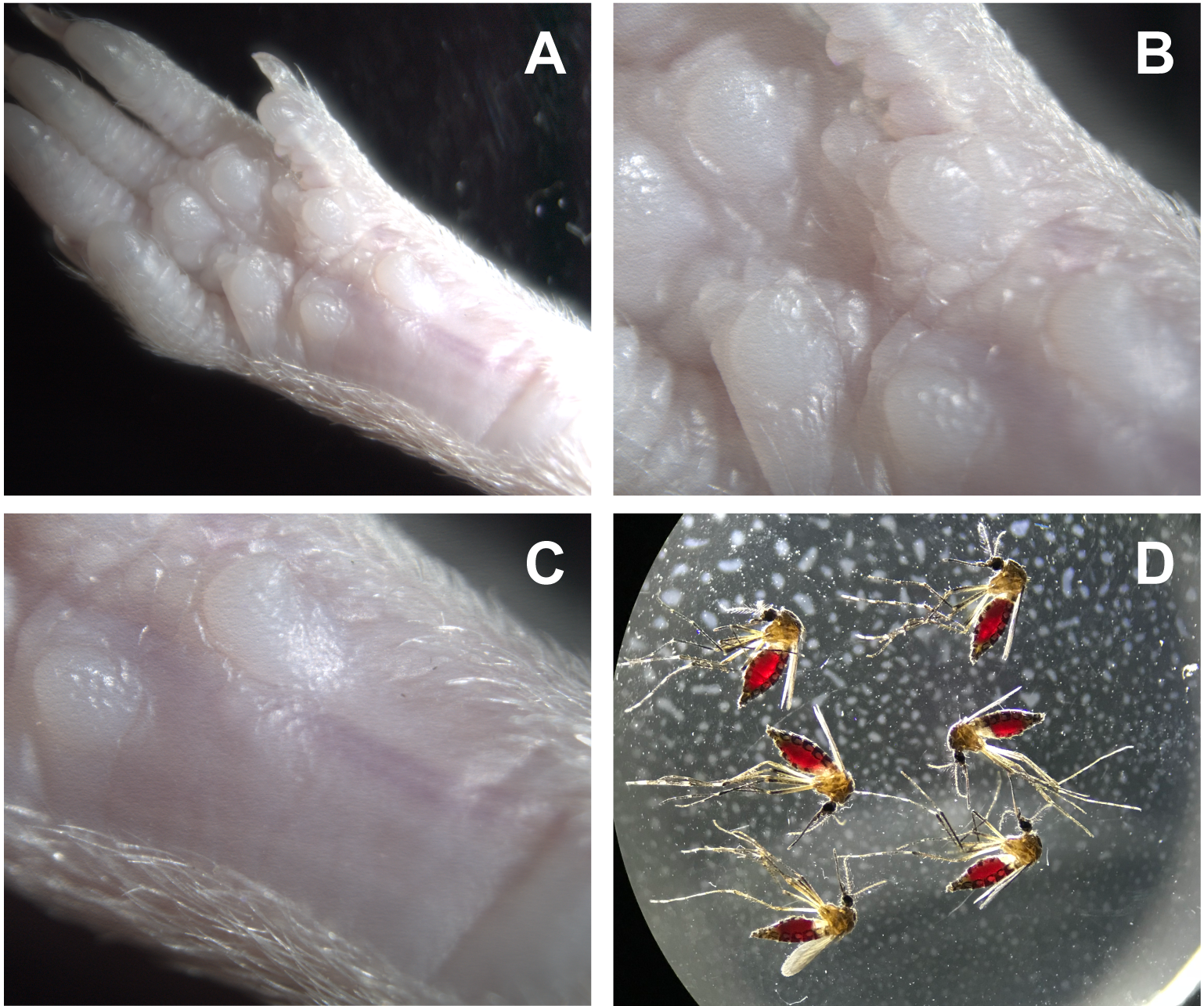

Supplement: S3 Fig — There is no evidence of injury or bleeding into the tissues after 3 mosquito bites on each footpad. (TIF) [file pntd.0006439.s003.tif]
