# Supplementary material for: Mosquito saliva alone has profound effects on the human immune system
Source: PLoS Negl Trop Dis. 2018 May 17;12(5):e0006439. doi: 10.1371/journal.pntd.0006439 (PMC5957326; doi:10.1371/journal.pntd.0006439)
Supplement: S1 Table — Mice are listed according to experimental group, with mouse ID, sex, and human CD45+ engraftment levels given. (DOCX) [file pntd.0006439.s004.docx]

Table S1: Humanized Mice Used in This Study

| **Earlier Studies** | | | | | | |
| --- | --- | --- | --- | --- | --- | --- |
| **Time Point** | **Group** | **Mouse ID** | **Sex** | **Engraftment (%)** | |  |
| 12 hours | Bite | 411 | Female | 87 | |  |
| 12 hours | Bite | 416 | Female | 43 | |  |
| 12 hours | Bite | 417 | Female | 43 | |  |
| 12 hours | Bite | 418 | Female | 68 | |  |
| 12 hours | Bite | 419 | Female | 26 | |  |
| 12 hours | Bite | 420 | Female | 35 | |  |
| 12 hours | Control | 393 | Female | 48 | |  |
| 12 hours | Control | 394 | Female | 55 | |  |
| 12 hours | Control | 408 | Female | 56 | |  |
| 12 hours | Control | 412 | Female | 68 | |  |
| 24 hours | Bite | 538 | Female | 72 | |  |
| 24 hours | Bite | 539 | Female | 74 | |  |
| 24 hours | Bite | 540 | Female | 59 | |  |
| 24 hours | Bite | 541 | Female | 74 | |  |
| 24 hours | Bite | 542 | Female | 77 | |  |
| 24 hours | Control | 427 | Female | 60 | |  |
| 24 hours | Control | 428 | Male | 54 | |  |
| 24 hours | Control | 468 | Female | 53 | |  |
| 24 hours | Control | 544 | Male | 61 | |  |
| 24 hours | Control | 545 | Male | 72 | |  |
| 48 hours | Bite | 18 | Male | 60 | |  |
| 48 hours | Bite | 39 | Female | 50.37 | |  |
| 48 hours | Bite | 40 | Female | 49.6 | |  |
| 48 hours | Bite | 41 | Female | 65.09 | |  |
| 48 hours | Bite | 45 | Female | 54 | |  |
| 48 hours | Bite | 48 | Male | 67.03 | |  |
| 48 hours | Control | 12 | Female | 35.26 | |  |
| 48 hours | Control | 16 | Male | 20.86 | |  |
| 48 hours | Control | 17 | Male | 39.7 | |  |
| 48 hours | Control | 22 | Female | 46.52 | |  |
|  | | | | | | |
| **Later Studies** | | | | | | |
| **Time Point** | **Group** | **Mouse ID** | **Sex** | **Engraftment (%)** |  |  |
| 6 hours | Bite | 312 | Female | 38.6 |  |  |
| 6 hours | Bite | 314 | Female | 41.3 |  |  |
| 6 hours | Bite | 315 | Male | 21.5 |  |  |
| 6 hours | Bite | 316 | Male | 30.7 |  |  |
| 6 hours | Bite | 342 | Male | 39.6 |  |  |
| 6 hours | Bite | 343 | Male | 46.9 |  |  |
| 6 hours | Bite | 797 | Female | 31.2 |  |  |
| 6 hours | Bite | 798 | Female | 29.2 |  |  |
| 6 hours | Bite | 800 | Female | 35.7 |  |  |
| 6 hours | Bite | 803 | Male | 30.5 |  |  |
| 6 hours | Bite | 806 | Male | 25.2 |  |  |
| 6 hours | Bite | 811 | Male | 33.8 |  |  |
| 6 hours | Control | 221 | Male | 27.9 |  |  |
| 6 hours | Control | 313 | Female | 22.8 |  |  |
| 6 hours | Control | 317 | Male | 23.3 |  |  |
| 6 hours | Control | 361 | Female | 21 |  |  |
| 6 hours | Control | 368 | Male | 27.3 |  |  |
| 6 hours | Control | 369 | Male | 22.4 |  |  |
| 6 hours | Control | 774 | Male | 10.5 |  |  |
| 6 hours | Control | 777 | Male | 10 |  |  |
| 6 hours | Control | 799 | Female | 21.6 |  |  |
| 6 hours | Control | 801 | Male | 13.4 |  |  |
| 6 hours | Control | 802 | Male | 17.9 |  |  |
| 6 hours | Control | 810 | Male | 22 |  |  |
| 24 hours | Bite | 139 | Male | 27 |  |  |
| 24 hours | Bite | 140 | Male | 27.3 |  |  |
| 24 hours | Bite | 141 | Male | 29.3 |  |  |
| 24 hours | Bite | 300 | Female | 21.8 |  |  |
| 24 hours | Bite | 301 | Female | 68.4 |  |  |
| 24 hours | Bite | 305 | Female | 22.2 |  |  |
| 24 hours | Bite | 370 | Female | 22.5 |  |  |
| 24 hours | Bite | 372 | Female | 15 |  |  |
| 24 hours | Bite | 373 | Male | 40 |  |  |
| 24 hours | Bite | 375 | Male | 18.4 |  |  |
| 24 hours | Bite | 381 | Male | 19.3 |  |  |
| 24 hours | Control | 142 | Female | 35.5 |  |  |
| 24 hours | Control | 143 | Female | 37.2 |  |  |
| 24 hours | Control | 144 | Female | 39.4 |  |  |
| 24 hours | Control | 307 | Male | 38.3 |  |  |
| 24 hours | Control | 308 | Male | 20.1 |  |  |
| 24 hours | Control | 310 | Male | 25.9 |  |  |
| 24 hours | Control | 371 | Female | 19.2 |  |  |
| 24 hours | Control | 374 | Male | 17 |  |  |
| 24 hours | Control | 376 | Male | 18.1 |  |  |
| 24 hours | Control | 380 | Male | 70 |  |  |
| 24 hours | Control | 382 | Male | 34 |  |  |
| 24 hours | Control | 384 | Female | 15 |  |  |
| 7 days | Bite | 350 | Female | 14.3 |  |  |
| 7 days | Bite | 352 | Female | 15.5 |  |  |
| 7 days | Bite | 354 | Male | 15.5 |  |  |
| 7 days | Bite | 355 | Male | 18.9 |  |  |
| 7 days | Bite | 356 | Male | 18.9 |  |  |
| 7 days | Bite | 358 | Male | 13.7 |  |  |
| 7 days | Bite | 581 | Male | 25 |  |  |
| 7 days | Bite | 593 | Male | 31.8 |  |  |
| 7 days | Bite | 618 | Female | 14.1 |  |  |
| 7 days | Bite | 619 | Female | 15.5 |  |  |
| 7 days | Bite | 620 | Female | 14.5 |  |  |
| 7 days | Bite | 622 | Male | 19.4 |  |  |
| 7 days | Control | 351 | Female | 14 |  |  |
| 7 days | Control | 362 | Male | 11.8 |  |  |
| 7 days | Control | 363 | Male | 15.6 |  |  |
| 7 days | Control | 364 | Male | 13.6 |  |  |
| 7 days | Control | 365 | Male | 12.6 |  |  |
| 7 days | Control | 367 | Male | 15.7 |  |  |
